# Supplementary material for: Experiences from six years of quality assured Model of End Stage Liver Disease (MELD) diagnostics
Source: PLoS One. 2021 Aug 26;16(8):e0254219. doi: 10.1371/journal.pone.0254219 (PMC8389365; doi:10.1371/journal.pone.0254219)

PLOS ONE

Experiences from Six Years of Quality Assured Model of End Stage Liver Disease (MELD) Diagnostics

--Manuscript Draft--

Manuscript Number:	PONE-D-20-34374
Article Type:	Research Article
Full Title:	Experiences from Six Years of Quality Assured Model of End Stage Liver Disease (MELD) Diagnostics
Short Title:	Experiences from Six Years of Quality Assured MELD Diagnostics
Corresponding Author:	Thorsten Kaiser<br>University Hospital Leipzig<br>Leipzig, GERMANY
Keywords:	MELD-score; Liver allocation; Quality- assured lab-MELD-Diagnostic; Liver transplant; End stage liver disease
Abstract:	Background<br>The model of end-stage liver disease (MELD) score was established for the allocation of liver transplants. The score is based on the medical laboratory parameters: bilirubin, creatinine and the international normalized ratio (INR). A quality-ensured laboratory MELD diagnostic was established, and the results from the first six years were analyzed.<br>Methods<br>We systematically investigated the validity of 7,270 MELD scores during a six-year period. The MELD score was electronically requested by the clinical physician using the laboratory system and calculated and specifically validated by the laboratory physician in the context of previous and additional diagnostics.<br>Results<br>In 2.7% (193 of 7,270) of the cases, MELD diagnostics did not fulfill the specified quality criteria. After consultation with the sender, 2.0% (145) of the MELD scores remained invalid for different reasons and could not be reported to the transplant organization. No cases of deliberate misreporting were identified. In 34 cases the dialysis status had to be corrected and there were 24 cases of oral anticoagulation with impact on MELD diagnostics.<br>Conclusion<br>Our quality assurance system for MELD diagnostics effectively prevented invalid MELD results and could be adopted by transplant centers to prevent diagnostic errors with possible adverse effects on organ allocation.
Order of Authors:	Pascal Hunold
	Thomas Berg
	Daniel Seehofer
	Robert Sucher
	Adam Herber
	Berend Isermann
	Joachim Thiery
	Thorsten Kaiser
Additional Information:	
**Question**	**Response**
**Financial Disclosure**<br><br>Enter a financial disclosure statement that describes the sources of funding for the	The funders had no role in study design, data collection and analysis, decision to publish, or preparation of the manuscript.

Powered by Editorial Manager® and ProduXion Manager® from Aries Systems Corporation

work included in this submission. Review the submission guidelines for detailed requirements. View published research articles from *PLOS ONE* for specific examples.<br><br>This statement is required for submission and **will appear in the published article** if the submission is accepted. Please make sure it is accurate.<br><br>**Unfunded studies**<br>Enter: *The author(s) received no specific funding for this work.*<br><br>**Funded studies**<br>Enter a statement with the following details:<br>• Initials of the authors who received each award<br>• Grant numbers awarded to each author<br>• The full name of each funder<br>• URL of each funder website<br>• Did the sponsors or funders play any role in the study design, data collection and analysis, decision to publish, or preparation of the manuscript?<br>• **NO** - Include this sentence at the end of your statement: *The funders had no role in study design, data collection and analysis, decision to publish, or preparation of the manuscript.*<br>• **YES** - Specify the role(s) played.<br><br>* typeset	
**Competing Interests**<br><br>Use the instructions below to enter a competing interest statement for this submission. On behalf of all authors, disclose any competing interests that could be perceived to bias this work—acknowledging all financial support and any other relevant financial or non-financial competing interests.<br><br>This statement **will appear in the published article** if the submission is accepted. Please make sure it is accurate. View published research articles from *PLOS ONE* for specific examples.	The authors have declared that no competing interests exist.

Powered by Editorial Manager® and ProduXion Manager® from Aries Systems Corporation

**NO authors have competing interests**<br><br>Enter: *The authors have declared that no competing interests exist.*<br><br>**Authors with competing interests**<br><br>Enter competing interest details beginning with this statement:<br><br>*I have read the journal's policy and the authors of this manuscript have the following competing interests: [insert competing interests here]*<br><br>* typeset	
**Ethics Statement**<br><br>Enter an ethics statement for this submission. This statement is required if the study involved:<br><br>• Human participants<br>• Human specimens or tissue<br>• Vertebrate animals or cephalopods<br>• Vertebrate embryos or tissues<br>• Field research<br><br>Write "N/A" if the submission does not require an ethics statement.<br><br>General guidance is provided below. Consult the submission guidelines for detailed instructions. **Make sure that all information entered here is included in the Methods section of the manuscript.**	N/A

**Format for specific study types**<br><br>**Human Subject Research (involving human participants and/or tissue)**<br>• Give the name of the institutional review board or ethics committee that approved the study<br>• Include the approval number and/or a statement indicating approval of this research<br>• Indicate the form of consent obtained (written/oral) or the reason that consent was not obtained (e.g. the data were analyzed anonymously)<br><br>**Animal Research (involving vertebrate animals, embryos or tissues)**<br>• Provide the name of the Institutional Animal Care and Use Committee (IACUC) or other relevant ethics board that reviewed the study protocol, and indicate whether they approved this research or granted a formal waiver of ethical approval<br>• Include an approval number if one was obtained<br>• If the study involved *non-human primates*, add *additional details* about animal welfare and steps taken to ameliorate suffering<br>• If anesthesia, euthanasia, or any kind of animal sacrifice is part of the study, include briefly which substances and/or methods were applied<br><br>**Field Research**<br><br>Include the following details if this study involves the collection of plant, animal, or other materials from a natural setting:<br>• Field permit number<br>• Name of the institution or relevant body that granted permission	
**Data Availability**<br><br>Authors are required to make all data underlying the findings described fully available, without restriction, and from the time of publication. PLOS allows rare exceptions to address legal and ethical concerns. See the PLOS Data Policy and FAQ for detailed information.	No - some restrictions will apply

A Data Availability Statement describing where the data can be found is required at submission. Your answers to this question constitute the Data Availability Statement and **will be published in the article**, if accepted.<br><br>**Important:** Stating 'data available on request from the author' is not sufficient. If your data are only available upon request, select 'No' for the first question and explain your exceptional situation in the text box.<br><br>Do the authors confirm that all data underlying the findings described in their manuscript are fully available without restriction?	
**Describe where the data may be found in full sentences. If you are copying our sample text, replace any instances of XXX with the appropriate details.**<br><br>• If the data are **held or will be held in a public repository**, include URLs, accession numbers or DOIs. If this information will only be available after acceptance, indicate this by ticking the box below. For example: *All XXX files are available from the XXX database (accession number(s) XXX, XXX.).*<br>• If the data are all contained **within the manuscript and/or Supporting Information files**, enter the following: *All relevant data are within the manuscript and its Supporting Information files.*<br>• If neither of these applies but you are able to provide **details of access elsewhere**, with or without limitations, please do so. For example:<br><br>*Data cannot be shared publicly because of [XXX]. Data are available from the XXX Institutional Data Access / Ethics Committee (contact via XXX) for researchers who meet the criteria for access to confidential data.*<br><br>*The data underlying the results presented in the study are available from (include the name of the third party*	Data cannot be shared publicly because of patient´s data protection. Data are available from the corresponding author for researchers who meet the criteria for access to confidential data.

and contact information or URL).<br>• This text is appropriate if the data are owned by a third party and authors do not have permission to share the data.<br><br>* typeset	
Additional data availability information:	

Manuscript
Click here to access/download;Manuscript;201016_Manuskript_quali_MELD

Experiences from Six Years of Quality Assured Model of End Stage Liver Disease (MELD) Diagnostics

Authors: Pascal Hunold[1], Prof. Dr. med. Thomas Berg[2], Prof. Dr. med. Daniel Seehofer[3], PD. Dr. med. univ. Robert Sucher[3], Adam Herber[2], Prof. Dr. med. Berend Isermann[1], Prof. Dr. med. Joachim Thiery[1], PD Dr. med. Thorsten Kaiser[1]*

[1]Institute of Laboratory Medicine, Clinical Chemistry and Molecular Diagnostics, University Hospital Leipzig, Germany

[2]Division of Hepatology, Clinic and Polyclinic for Gastroenterology, Hepatology, Infectious diseases and Pneumology, University Hospital Leipzig, Germany

[3]Department of Visceral, Transplant, Thoracic and Vascular Surgery, University Hospital Leipzig, Germany

Correspondence:

PD. Dr. med. Thorsten Kaiser

Institute of Laboratory Medicine, Clinical Chemistry and Molecular Diagnostics

University Hospital Leipzig

Paul-List-Str. 13-15

04103 Leipzig

Germany

Telephone: +49 341 9722200

Fax: +49 341 22209

E-mail: thorsten.kaiser@medizin.uni-leipzig.de

word count (text):	2457
word count (abstract):	200
references:	28
Figures/ tables:	4

Abstract

Background

The model of end-stage liver disease (MELD) score was established for the allocation of liver transplants. The score is based on the medical laboratory parameters: bilirubin, creatinine and the international normalized ratio (INR). A quality-ensured laboratory MELD diagnostic was established, and the results from the first six years were analyzed.

Methods

We systematically investigated the validity of 7,270 MELD scores during a six-year period. The MELD score was electronically requested by the clinical physician using the laboratory system and calculated and specifically validated by the laboratory physician in the context of previous and additional diagnostics.

Results

In 2.7% (193 of 7,270) of the cases, MELD diagnostics did not fulfill the specified quality criteria. After consultation with the sender, 2.0% (145) of the MELD scores remained invalid for different reasons and could not be reported to the transplant organization. No cases of deliberate misreporting were identified. In 34 cases the dialysis status had to be corrected and there were 24 cases of oral anticoagulation with impact on MELD diagnostics.

Conclusion

Our quality assurance system for MELD diagnostics effectively prevented invalid MELD results and could be adopted by transplant centers to prevent diagnostic errors with possible adverse effects on organ allocation.

Introduction

End stage chronic liver and metabolic diseases can only be effectively treated with orthotopic liver transplantation. [1] The allocation of liver grafts in many European and other countries is performed according to the laboratory model for end-stage liver disease (Lab-MELD) score. The Lab-MELD score is calculated based on three blood biomarkers: bilirubin, creatinine and the international normalized ratio (INR). [2] The MELD score predicts the three-month mortality rate for patients with end-stage liver disease.

Due to the importance of the MELD score for the further therapy options of the patient, a high validity of the diagnosis is essential. There are known limitations of the MELD score, which are further engraved by preanalytic limitations. [3] [4] [5]

Dialysis influences the serum creatine concentration. Therefore, for calculation of the MELD score the creatine is set as 353.6 μmol/l (4.0 mg/dl), if at least two dialyses were performed within the prior week or if a patient received 24 hours of continuous veno-venous hemodialysis (CVVHD) within the prior week. [6]

The lab-MELD score replaced the previously applied, more clinically-based organ allocation system in Germany in 2006 to reduce the mortality among patients on the waiting list. [2] [7] [8] [9] [10] [11] Countries in the Eurotransplant region (such as Austria, Belgium, Croatia, Germany, Hungary, the Netherlands, Luxembourg and Slovenia) use the MELD score for the allocation of organs. The United Network for Organ Sharing, responsible for organ allocation in the United States of America, uses the MELD Na score for patients with an initial MELD score of at least 11 for allocation. [12] In addition to the three MELD parameters, since 2016 the sodium concentration in serum is included in the MELD Na score. [13]

For a minority of the patients on the waiting list—for example, patients with hepatocellular carcinoma or primary sclerosing cholangitis and special complications—the transplantation guidelines define specific exceptions. In these cases, an individual match-MELD score under

consideration of the waiting time is calculated and replaces the lab-MELD score for the allocation process. [7]

In 2012, irregularities in MELD diagnostics were reported in different transplant centers in Germany. [14] These violations contributed to a decrease in organ donation in Germany. As a potential result, the shortage of donor organs was further aggravated. [15] [16]

To improve the validity of the MELD diagnostics, we implemented a specific quality-ensured lab-MELD diagnostic in 2012 (Figure 1). This study aimed to prove the effectiveness of our quality assurance procedure to avoid misclassifications of patients with indication for a liver transplant.

Methods

The lab-MELD validation and reporting system (Figure 1) was implemented in the laboratory information system in November 2012 by the Institute of Laboratory Medicine. Before the implementation of the specific quality assurance of MELD diagnostics, there was an interdisciplinary discussion at the University Hospital Leipzig. The implementation was unanimously decided by the Institute of Laboratory Medicine; the Department of Visceral, Transplantation-, Vascular and Thoracic Surgery; the Section of Hepatology; the Department of Gastroenterology and Rheumatology; and the Hospital Management Board.

Each MELD diagnostic was reported in a specific document for further transfer of the data to Eurotransplant. The Institute for laboratory medicine, as an independent institution, supervised the preanalytical and clinical conditions and the plausibility of the results for every patient (Figure 1). The MELD diagnostic was requested electronically by the clinicians using the laboratory information system. This request included the specification of hemodialysis and anticoagulation status.

Lab-MELD diagnostic was performed when indicated for clinical diagnosis of patients in evaluation for orthotopic liver transplantation. There was no need for additional blood takings for this study. If necessary, additional clinical information had been collected from the electronic patient records. All methods were carried out in accordance with existing, relevant guidelines and regulations. The approach was approved by the Faculty of Medicine ethics committee of the Leipzig University.

The diagnostic workup of the MELD diagnostic required quality assurance of the clinical diagnostic process and ethics committee agreed to the usage of these results also for scientific investigation without the specific informed consent of the patients.

Statistics

The data for all three analytes—serum bilirubin, serum creatinine and the INR—and the resulting MELD score were analyzed by mean values, standard deviations and the Chi-squared test using SPSS software, version 24.0 (IBM, Ehningen, Germany), and Microsoft Excel 2010 (Microsoft Corporation, Redmond, USA).

Results

From November 2012 to October 2018, 7,270 lab-MELD (3.3 per day or 23.4 per week) diagnostics were performed. Table 1 summarizes the characteristics of cases of 1,494 different patients that required MELD diagnostics.

	Women	Men	Total	p – Value
**MELD- diagnostics (number of different patients)**	2633 (553)	4637 (941)	7270 (1494)	
**Bilirubin (Range) [µmol/L]**	34.8 (2.2 - 1096,8) n= 2625	31.6 (2.6 – 785.4) n= 4627	31.7 (2.2 -1096.8) n= 7252	0.006
**Creatinin (Range) [µmol/L]**	81 (20 – 610) n= 2628	89 (21 – 786) n= 4628	86 (20 – 786) n= 7256	< 0.001
**INR (Range)**	1.4 (0.8 – 9) n= 2589	1.3 (0.8 – 12.9) n= 4583	1.3 (0.8 – 12.9) n= 7172	< 0.001
**Dialysis**	253 (9.7%)	305 (6.6%)	558 (7.7%)	< 0.001

Table 1: Summary of the characteristics of cases that required MELD diagnostic from November 2012 until October 2018 within the University Hospital Leipzig. Only valid results that could be reported to the transplant organization are displayed.

Specific medical laboratory validation of MELD diagnostics

During the first six years, 7,270 MELD diagnostics were performed. In 97.3% of the cases (7,077), a valid MELD score could be reported. In total 524 patients received anticoagulation treatment. Of those, 2 patients were treated with Fondaparinux, 488 with heparin, 33 with a Vitamin K antagonist and 1 patient with a suggested intake of Apixaban, a direct oral FXa

inhibitor). These MELD scores were separately validated for plausibility by comparing the current results to previous results without anticoagulation in the clinical context and INR was unchanged.

In the remaining 193 cases the initially requested MELD score could neither be validated nor reported to the transplant organization for different reasons. These cases are illustrated in detail in Figure 2.

After individual investigation, 48 valid MELD scores could be provided. In 30 of these cases, the indicated positive dialysis status could not be confirmed by checking the patients record and consulting the sender of the samples. In 8 cases dialysis was performed; however, but not as defined in the dialysis criteria within the week; CCVHD or CVHD were not performed for the demanded time. [17] For 22 cases the lab data were not congruous with performance of dialysis. After consultation with the clinical physicians a mistake during order creation was evaluated. One patient received Albumin dialysis. This method does not equal a kidney replacement method. Dialysis was changed in these 30 cases to “no dialysis” resulting in lower MELD scores (median 33.0 [before] vs. 22.5 [after]). After this correction, the results were approved and reported to the transplant organization.

In four cases, the dialysis status was incorrectly indicated as negative when the diagnostic was requested. After consulting the clinics and the dialysis unit, the status was corrected to “yes.”

In the remaining 145 cases, the MELD score could not be reported at all to the transplant organization due to different reasons (Figure 2).

In 85 cases, the MELD score could not be determined or reported to the transplant office because of major preanalytical errors with a probable impact on the resulting MELD score (Table 2).

Preanalytical errors leading to invalid MELD diagnostic	
**Missing material (either blood serum or citrate blood)**	44
**Underfilling with inadequate mixing ratio citrate vs. blood**	7
**Confusion of patients in the process of blood withdrawal**	7
**Incorrect labeling of monovettes (wrong material)**	6
**Prolonged preanalytical period (>4h)**	5
**MELD requested for children under the age of 12 years (PELD necessary)**	5
**Not all required parameters (INR, creatinine, bilirubin) requested**	4
**Citrate blood clotted**	3
**Late laboratory entrance**	2
**Ascites was sent in instead of blood serum**	1
**Lithium heparin instead of blood serum**	1

Table 2: Table with preanalytical errors leading to not reporting MELD score

In these cases, a new blood sample had to be taken to guarantee a valid MELD score. In four cases, the sender did not state the status of dialysis and the status could not be ascertained. In seven specimens, the citrate tubes were significantly underfilled with a probable impact on INR and were not usable for a valid MELD diagnostic, as underfilling can lead to false high INR values. In seven cases, there was a confusion of patients' identity during blood withdrawal, which was identified during medical validation. In five cases, a MELD score was requested for children aged 1–6 years but was not reported as the MELD score is only validated for adolescents and adults aged 12 years and above. In these cases,

the clinical physician was informed and the pediatric end-stage liver disease (PELD) score was used. [18]

In 24 cases with anticoagulation, there was a significant influence on INR and consequently the MELD score. In these cases, the INR (in median 2.65) and MELD scores (in median 21.0) were significantly higher compared to the rest of the patients ($p < 0.001$ and $p = 0.014$, respectively). Seventeen of these cases were due to the intake of Vitamin K antagonists and were already stated in the MELD diagnostic request. In the process of validation by the laboratory, one case of the intake of Vitamin K antagonists was unraveled, which was not indicated before. Another four cases of Rivaroxaban intake, one of Apixaban intake (both direct oral factor Xa-inhibitors), one other not specified oral antagonist and one case of a massive overdose of heparin, which relevantly inhibited the prothrombin time test, was identified by the laboratory and could therefore not be reported.

Invalid clotting tests in patients who required MELD diagnostics

In 42 of the 7,270 (0.57%) cases, the automatized clotting tests did not display results because of the massive lack of coagulation factors in advanced liver failure. In 10 cases, a result could be achieved through manual interpretation of the clotting curve by the laboratory physician. The INR in these patients was between 3.3 and 9 with a resulting MELD score from 27 to 40. In one of these cases, the INR result was not plausible in the context of the pre-results for unknown reasons. The follow-up measurements of the patient resulted in plausible results.

In 31 specimens of 20 patients, a valid determination of the prothrombin time/INR was not possible. In these cases, an additional sample was requested, which was sent in in 24 cases. In 16 of these cases, a prothrombin time/INR diagnostic was possible in the median after 23.9 h (4.4–48.2 h). Ten of these cases had received a prior substitution of coagulation factors or fresh frozen plasma.

Importantly, 13 of these 21 patients (62%) died within 2 days (0–88 days) after the first undeterminable prothrombin time/INR and 4 patients received liver transplantation within 6.5 days (0–16 days).

Discussion

We report for the first time experiences with a specific laboratory quality assurance system to improve the validity of MELD diagnostic. To the best of our knowledge, our transplant center was the first to implement such a specific quality assurance system for MELD diagnostics in the clinical routine.

The majority of the MELD diagnostics (97.3%) passed the process of analysis and validation without any doubt of validity. However, the MELD diagnostic can be complex as many regulatory requirements exist. In this study, we identified different deviations from valid MELD diagnostics.

The clinical physicians stated the dialysis status during the laboratory order creation. The status was checked for plausibility in the context of the other laboratory results during medical validation. However, in some cases, the given dialysis status did not fulfill the official criteria. The unawareness of the regulations of unacquainted interns as well as inadvertent mistakes during order creation lead to an incorrect stated status. After medical validation by the laboratory physician, no cases of deliberate misreporting were identified. The transplantation office reconfirmed each of the cases if the dialysis status met the official dialysis criteria by checking the dialysis protocols before the admittance to the transplant organization.

The intake of oral anticoagulation also influences the MELD score by raising the INR. [19] [20] [21] The deliberate or unintentional concealment of the intake of these drugs could lead to elevated MELD scores and the prioritization of those patients in the process of allocation. During the medical validation, the stated anticoagulation and the INR were validated in the context of other biomarkers for liver function, such as albumin, cholinesterase and bilirubin.

Furthermore, the INR was compared to previous results and analyzed for implausible constellations. INR results indicating the use of anticoagulation were not reported. However, from the laboratory parameters alone, it is not possible to exclude the use of anticoagulation in patients with minor changes in INR.

A very relevant and not conclusively solved problem is the handling of not determinable INR in the majority of cases due to the lack of fibrinogen. In the case of an extreme lack of coagulation factors, the prothrombin time and the calculated INR are dependent on the time used to determine the clotting reaction. There were 21 patients with no clotting within 315 sec. This maximum waiting time for the coagulation reaction to occur is individually defined by the medical laboratories. In the case of a tremendous lack of fibrinogen, independently from other clotting factors concentrations, no reaction will be detectable at all and the laboratory calculates an INR based on the calibration curve at the maximum of the waiting time. The calculation of the INR and the submission to the transplant organization with a longer waiting time results in extremely high MELD scores and increased chances to receive a transplant. The objectives of Eurotransplant state that in these cases, an Eurotransplant liver intestine advisory committee (ELIAC) auditor could determine an INR equivalent using the highest prothrombin time from the conversion table provided by the laboratory since the tables differ among the laboratories and manufacturers. [17] In our experience, the procedure is inconsistent and needs further clarification of the regulations for Eurotransplant as well as United Network for Organ Sharing (UNOS). For unknown reasons, the advisory committee decided only to accept the last determinable INR that resulted in a lower MELD score compared to the results obtained from the maximum of the INR-conversion table. This is highly relevant, as many patients with an undeterminable INR had to be supplemented with clotting factors and died within a short time (13 of 21 patients [62%] in our study).

Most problems leading to an invalid result in laboratory measurements occurred in the context of the preanalytical phase. [22] The medical validation of the MELD score could help to reveal and reduce the problems in the preanalytic phase of the diagnostic process. The

confusion of patients' identity is a major problem leading to inaccurate MELD scores. We identified seven cases.

Another relevant preanalytical problem could be time-dependent changes in the specimen. The delayed laboratory analysis of citrate blood could influence the INR leading to a falsely elevated MELD score. [23] In our study, no hints of outdated samples were detected.

Another limitation of MELD Diagnostics cannot be solved by our system. There is already evidence that the measurement methods have an impact on the concentration of the analytics and the MELD score. [24] [25] [26] [27] [28] Therefore, methods used for the determination of the parameters needed to calculate the MELD score should be specified to guarantee an equitable allocation.

As MELD diagnostics is of life determinable consequences, a valid determination needs to be assured. We were able to show the positive effects of specific quality assurance of MELD diagnostics. Advantages of our approach are an identification and prevention of invalid MELD diagnostics in a relevant number of cases with invalid dialysis status, impact of anticoagulation therapy and further preanalytical errors. Furthermore, our system allows to preserve blood samples for quality assurance and remeasurements if indicated. A broad implementation of a laboratory quality assurance system in transplant centers could help to guarantee that organ allocation is directly related to the severity of the liver disease.

References

1 Bachir NM, Larson AM. Adult liver transplantation in the United States. Am. J. Med. Sci. 2012; 343:462–469.

2 Kamath PS, Kim WR. The model for end-stage liver disease (MELD). Hepatology 2007; Mar;45(3):797-805.

3 Sacleux SC, Samuel D. A Critical Review of MELD as a Reliable Tool for Transplant Prioritization. Semin Liver Dis. 2019;39(4):403-413. doi:10.1055/s-0039-1688750

4 Locke JE, Shelton BA, Olthoff KM, et al. Quantifying Sex-Based Disparities in Liver Allocation [published online ahead of print, 2020 May 20]. JAMA Surg. 2020;e201129. doi:10.1001/jamasurg.2020.1129

5 Tripodi A, Chantarangkul V, Primignani M, et al. The international normalized ratio calibrated for cirrhosis (INR(liver)) normalizes prothrombin time results for model for end-stage liver disease calculation. Hepatology. 2007;46(2):520-527. doi:10.1002/hep.21732

6 Eurotransplant Manual- version 5.11. February 2019, 11

7 German Medical Association. Guidelines according to organ transplantation. § 16 Section 1 Sentence 1, No 2. and 5. TPG. Dtsch Arztebl 2013 110 (6):A-241 /. B-225 /. C-225.

8 Durand F, Valla D. Assessment of the prognosis of cirrhosis: Child-Pugh versus MELD. J Hepatol 2005; 42:S100-7.

9 Schlitt HJ, Loss M, Scherer MN, Becker T, Jauch K, Nashan B, et al. Current developments in liver transplantation in Germany: MELD-based organ allocation and incentives for transplant centres. Z Gastroenterol 2011; 49:30–38.

10 Asrani SK, Kim WR. Model for end-stage liver disease: end of the first decade. Clin Liver Dis 2011; 15:685–698.

11 Luo, X, Leanza, J, Massie, AB, et al. MELD as a metric for survival benefit of liver transplantation. Am J Transplant. 2018; 18: 1231– 1237.

12 OPTN Policy 9: Allocation of livers and liver-intestines. 01.10.2019. 169.

13 Biggins SW, Kim WR, Terrault NA, Saab S, Balan V, Schiano T, Benson J, Therneau T, Kremers W, Wiesner R. Evidence-based incorporation of serum sodium concentration into MELD. Gastroenterology. 2006;130:1652-1660.

14 German Medical Association 2013. Presentation of the test results of all 24 liver transplant programs in Germany (article in German). Available at: http://www.bundesaerztekammer.de/downloads/2013-09-04_Bericht_PK_UK_2012-2013_4.pdf. Accessed: 04 Apr 2019.

15 E. Tackmann, S. Dettmer: Acceptance of post-mortem organ donation in Germany. Representative cross-sectional study. Anaesthesist 2018.67:118-125.

16 Eurotransplant Statistics Report Library. Deceased donors used, by year, by donor country. http://statistics.eurotransplant.org/index.php?search_type=donors+deceased&search_organ=liver&search_region=All+ET&search_period=by+year&search_characteristic=&search_text=. Accessed: 04 Apr 2019.

17 Eurotransplant Manual- version 5.11. February 2019, 46-47.

18 Eurotransplant Manual- version 5.11. February 2019, 19.

19 D.M. Adcock, R. Gosselin: Direct Oral Anticoagulants (DOACS) in the Laboratory: 2015 Review. Thromb Res. 2015 Jul; 136 (1): 7-12.

20 Mahmut Tobu, Omer Iqbal, Debra Hoppensteadt, Brian Neville, Harry L. Messmore, Jawed Fareed: Anti-Xa and anti-IIa drugs alter international normalized ratio measurements: potential problems in the monitoring of oral anticoagulants. Clin Appl Thromb Hemost. 2004 Oct;10(4):301-9

21 Gu- Ha Alai, Han- Yu Deng, Gang Li, Jun Luo, Lun- Xu Liu, Yi- Dan Lin: The influence of heparin on coagulation function of patients undergoing video- assisted major thoracic surgery. J Thorac Dis. 2018 Apr;10(4):2288-2294.

22 Paolo Carraro, Mario Plebani: Errors in a Stat Laboratory: Types and Frequencies 10 Years Later. Clinical Chemistry Jul 2007, 53 (7) 1338-1342

23 Johanna H.H. van Geest- Daalderop, Andre B. Mulder, Leandra J.K. Boonman- de Winter, Martha M.C.L. Hoekstra, Anton M.H.P. van den Besselaar: Preanalytical variables and off- site blood collection: influences on the results of the prothrombin time/international normalized ratio test and implications for monitoring of oral anticoagulant therapy. Clin Chem. 2005 Mar;51(3):561-8.

24 Kaiser T, Kinny-Koster B, Bartels M, Parthaune T, Schmidt M, Thiery J. (2014): Impact of different creatinine measurement methods on liver transplant allocation. PLoS ONE 9(2).

25 Kaiser T, Kinny-Köster B, Gnewuch C, Karailieva D, Kiehntopf M, Kessler A, Ritter-Sket C, Schmidt M, Brand K, Thiery J, Lichtinghagen R. Limited comparability of creatinine assays in patients with liver cirrhosis and their impact on the MELD score. Practical Laboratory Medicine 2017;8: 41–8.

26 Meijer P, Kynde K, van den Besselaar A, Van Blerk M, Woods T: International normalized ratio (INR) testing in Europe: between- laboratory comparability of test results obtained by Quick and Owren reagents. Clin Chem Lab Med 2018; 56(10): 1698-1703.

27 Gu D, Wang Y, Ren B, Wang L, Zhang K, Yuan Y: Comparison of Three Routine Methods of the Measurement of Serum Bilirubin in a China Laboratory. Clin Lab 2018 Sept 1; 64(9):1485-1490.

28 Hoste L, Deiteren K, Pottel H, Callewaert N, Martens F: Routine serum creatinine measurements: how well do we perform?. BMC Nephrol. 2015 Feb 14;16-21

Author contributions

**PH**	**designed research/study, performed research, analyzed data, wrote paper**
**TB**	study concept and design, analysis and interpretation of data, critical revision of the manuscript
**DS**	study concept and design, analysis and/or interpretation of data, critical revision of the manuscript
**RS**	study concept and design
**AH**	study concept and design
**BI**	study concept and design, analysis and/or interpretation of data, critical revision of the manuscript
**JT**	study concept and design, analysis and/or interpretation of data
**TK**	designed research/ study, performed research, analyzed data, wrote paper

Funding sources

The authors received no specific funding for this work.

Conflict of Interest

not applicable

Figure Click here to access/download;Figure;Figures_PLOS.docx

Figure 1: Flow sheet of quality approved lab-MELD Diagnostic at the University Hospital Leipzig

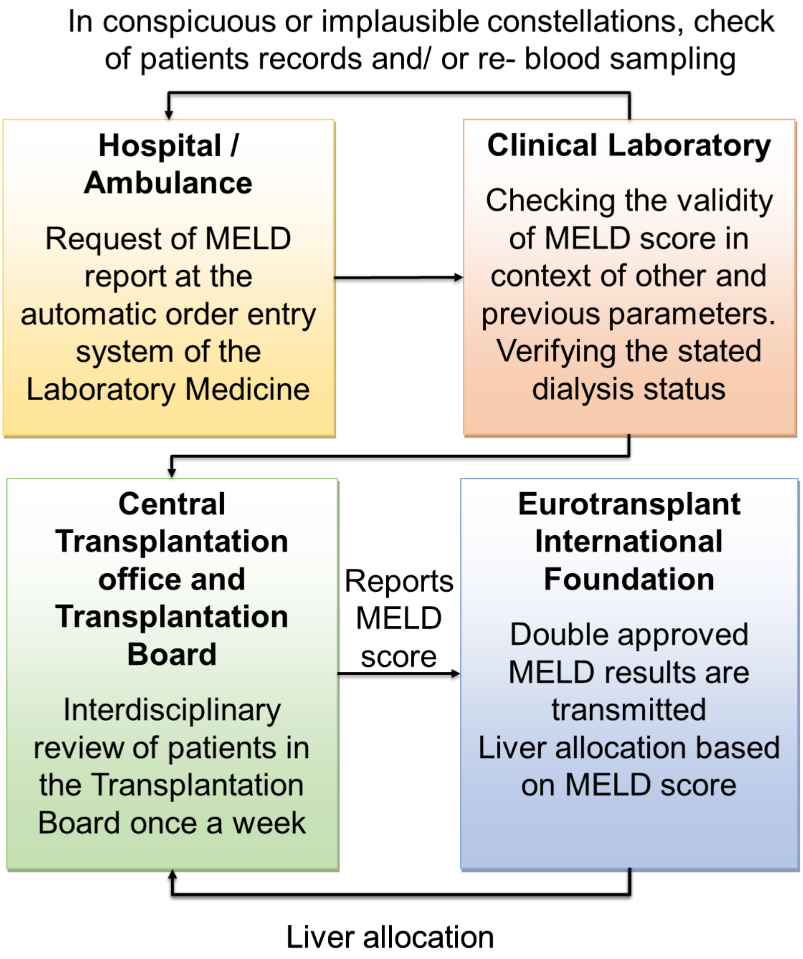

Figure 2: Summary of the requested MELD scores and the results of the laboratory medical validation. Anticoagulation might be indicated within patients with liver diseases. However, INR must be unchanged for valid MELD- diagnostics. In 25 cases, treatment with anticoagulation had influence on the INR and consequently on the MELD score. Therefore, a validation was not possible.
***** Dialysis status or interval did not fulfill the official criteria. However, no cases of deliberate misreporting were identified.

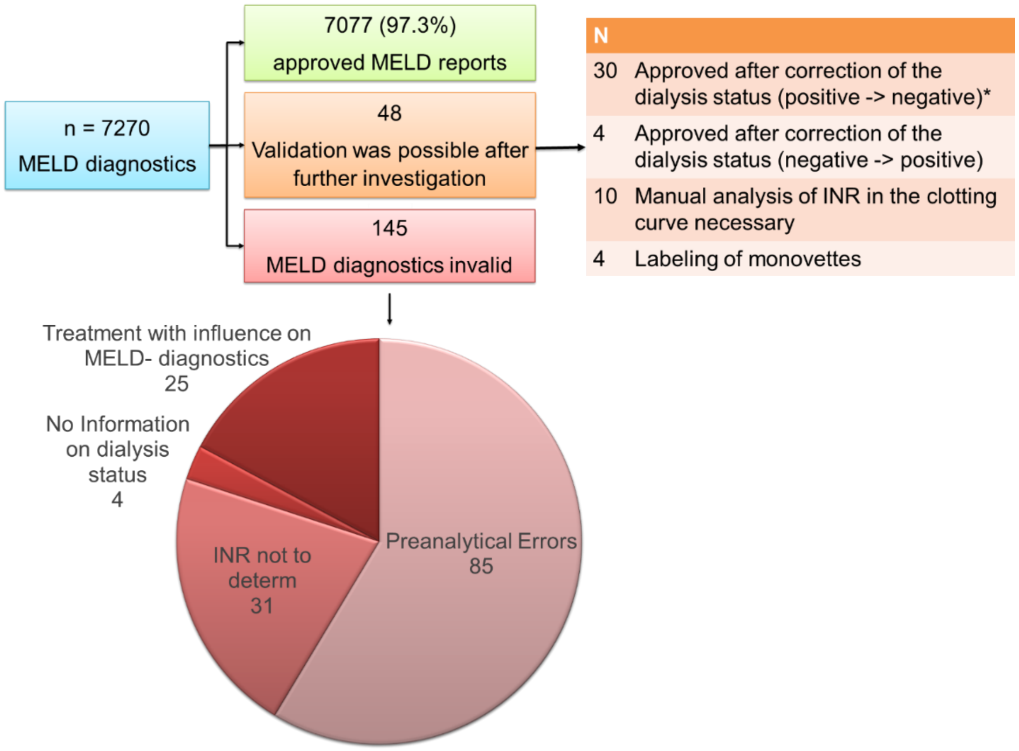

Supplement: Attachment — Submitted filename: PONE-D-20-34374_reviewer.pdf [file pone.0254219.s001.pdf]
