# Supplementary material for: Experiences from six years of quality assured Model of End Stage Liver Disease (MELD) diagnostics
Source: PLoS One. 2021 Aug 26;16(8):e0254219. doi: 10.1371/journal.pone.0254219 (PMC8389365; doi:10.1371/journal.pone.0254219)
Supplement: Attachment — Submitted filename: 210218_PLOSONE_rebuttal_letter_PHTK_final.docx [file pone.0254219.s002.docx]

PLOS ONE
Journal Requirements:

When submitting your revision, we need you to address these additional requirements.

 1. Please ensure that your manuscript meets PLOS ONE's style requirements, including those for file naming. The PLOS ONE style templates can be found at <https://journals.plos.org/plosone/s/file?id=wjVg/PLOSOne_formatting_sample_main_body.pdf> and <https://journals.plos.org/plosone/s/file?id=ba62/PLOSOne_formatting_sample_title_authors_affiliations.pdf>

**Thank you for the information. We corrected the formatting according to your regulations within the whole manuscript.**

 2.We note that you have indicated that data from this study are available upon request. PLOS only allows data to be available upon request if there are legal or ethical restrictions on sharing data publicly. For information on unacceptable data access restrictions, please see <http://journals.plos.org/plosone/s/data-availability#loc-unacceptable-data-access-restrictions>.

 In your revised cover letter, please address the following prompts:

 a) If there are ethical or legal restrictions on sharing a de-identified data set, please explain them in detail (e.g., data contain potentially identifying or sensitive patient information) and who has imposed them (e.g., an ethics committee). Please also provide contact information for a data access committee, ethics committee, or other institutional body to which data requests may be sent.

 b) If there are no restrictions, please upload the minimal anonymized data set necessary to replicate your study findings as either Supporting Information files or to a stable, public repository and provide us with the relevant URLs, DOIs, or accession numbers. Please see <http://www.bmj.com/content/340/bmj.c181.long> for guidelines on how to de-identify and prepare clinical data for publication. For a list of acceptable repositories, please see <http://journals.plos.org/plosone/s/data-availability#loc-recommended-repositories>.

 We will update your Data Availability statement on your behalf to reflect the information you provide.

**Thank you, we addressed this topic in our revised cover letter.**

 3.Thank you for stating the following financial disclosure:

 "The funders had no role in study design, data collection and analysis, decision to publish, or preparation of the manuscript."

At this time, please address the following queries:

a. Please clarify the sources of funding (financial or material support) for your study. List the grants or organizations that supported your study, including funding received from your institution.
b. State what role the funders took in the study. If the funders had no role in your study, please state: “The funders had no role in study design, data collection and analysis, decision to publish, or preparation of the manuscript.”
c. If any authors received a salary from any of your funders, please state which authors and which funders.
d. If you did not receive any funding for this study, please state: “The authors received no specific funding for this work.”

Please include your amended statements within your cover letter; we will change the online submission form on your behalf.

 4. Please ensure you have discussed any potential limitations of your study in the Discussion.

**To best of our knowledge, we discussed all known limitations.**

 5. In your Methods section, please provide additional information about the medical data/samples collected and the demographic details of the human subjects. Please ensure you have provided sufficient details to replicated the analyses such as: a) a description of any inclusion/exclusion criteria that were applied to the medical records/samples,

**We extended the inclusion and exclusion criteria section on page 8, line 142f.**

b) a table of relevant demographic details

**We added the demographic detail „age“ in Table 1, row 3.**

c) a description of how the medical records/samples were collected.

**We completed the information on page 7, line 126f.**

 6. In the ethics statement in the manuscript and in the online submission form, please provide additional information about the patient records/samples used in your retrospective study, including: a) whether all data were fully anonymized before you accessed them; b) the date range (month and year) during which patients' medical records/samples were accessed.

**We added the detailed information on page 8, line 142ff.**

Additional Editor Comments:

I find the manuscript of some value, even though one reviewer recommend rejection.

Anyhow, I’ll allow a major revision.

You should emphasize more on the laboratory errors around reporting the MELD score.
**Thank you very much for this important aspect. We are aware and discussed the laboratory errors in context of indeterminable INR values and in context of different measuring methods in between different laboratories. We expanded the discussion of preanalytical errors in following sections: Figure 1, page 7 line 113ff, page 12 line 211ff, page 14 lines 254ff.**

Being in a laboratory myself, I wonder why you system does not automatically request all three parameters when MELD is ordered

**Thank you, we totally agree. As you suspected, the laboratory information system automatically requests all needed methods and materials. We reevaluated the four cases of missing MELD parameters. The investigation revealed that one material was missing. For clarity, we now assigned these cases to „material error“.**

and why it not automatically substitute MELD with PELD when the age is <12yrs?

**Thank you, this is a relevant point. When we designed our lab-MELD framework, we discussed this aspect. However, due to the very limited number of patients requiring a PELD (only about 1 per year), the effort appeared too high in relation to the expected benefit. We address this point on page 12 lines 210ff.**

In table 2, several of the errors seems belonging to the same group, missing material, wrong material, lithium instead of serum.

**Thanks for this suggestion to increase readability and clarity. We summarized these points as „material error“ in table 2.**

“Confusion of patients in the process of blood withdrawal” is that the code for incorrect ID?

**Thank you for this aspect. Exactly, we meant a lab-ID requested for another patient. As we have a highly automatized laboratory with** **seamless barcode identification of every probe, the patient’s confusion could only take place at the station. We specified this point in table 2.**

“Incorrect labeling of monovettes (wrong material)” is that patient label og a producer error?

**The material-ID did not match the actual material. We renamed this point in table 2.**

Some details concerning the analytic methods would be nice, calibration CV, QC.

**Thank you for these important laboratory specific aspects. We extended this section (page 7 lines 127ff).**

SPSS is not originating in Ehningen, the correct citation should be “IBM Corp. Released 2016. IBM SPSS Statistics for Windows, Version 24.0. Armonk, NY: IBM Corp”, or some abbreviation hereof.

**Thank you, we corrected this information on page 8 line 150.**

Reviewers' comments:

Reviewer's Responses to Questions

Comments to the Author

1. Is the manuscript technically sound, and do the data support the conclusions?

The manuscript must describe a technically sound piece of scientific research with data that supports the conclusions. Experiments must have been conducted rigorously, with appropriate controls, replication, and sample sizes. The conclusions must be drawn appropriately based on the data presented.

Reviewer #1: Partly

Reviewer #2: Yes

2. Has the statistical analysis been performed appropriately and rigorously?

Reviewer #1: Yes

Reviewer #2: Yes

3. Have the authors made all data underlying the findings in their manuscript fully available?

The [PLOS Data policy](http://www.plosone.org/static/policies.action#sharing) requires authors to make all data underlying the findings described in their manuscript fully available without restriction, with rare exception (please refer to the Data Availability Statement in the manuscript PDF file). The data should be provided as part of the manuscript or its supporting information, or deposited to a public repository. For example, in addition to summary statistics, the data points behind means, medians and variance measures should be available. If there are restrictions on publicly sharing data—e.g. participant privacy or use of data from a third party—those must be specified.

Reviewer #1: Yes

Reviewer #2: No

4. Is the manuscript presented in an intelligible fashion and written in standard English?

PLOS ONE does not copyedit accepted manuscripts, so the language in submitted articles must be clear, correct, and unambiguous. Any typographical or grammatical errors should be corrected at revision, so please note any specific errors here.

Reviewer #1: Yes

Reviewer #2: Yes

5. Review Comments to the Author

Reviewer #1: The paper is reviewing the experience of a quality assurance system of validation and report of MELD score. All the results shows the good performance of this system to detect invalid MELD scores due to different reasons.
I think these results should be submitted to a different type of journal more focused in Clinical Chemistry and/or Laboratory
In the discussion , it should be emphasized to interpret this MELD score in the clinical context of patients . The clinician must understand , before sending the data , the intrinsic limitations of MELD . This score is NOT design for patient under dialysis or under anticoagulation therapy .All this scores must be validated before and/or after the results of the laboratory to the final use of allocation of patients in a waiting list . There is a some exceptions for MELD and also some limitations that make essential the evaluation of the physician. All this aspects must be discussed in the paper.

**Thank you for this very relevant aspect. We totally agree, that the clinicians have to understand MELD diagnostics, including indications, interpretation and regulation. However, to our experience, the specific knowledge of some clinicians is limited and our framework and the resulting feedback improved awareness. We included further information on this point page 5 lines 68ff, page 7 lines 118ff, as well as in the discussion page 14 lines 254ff. “Our verification algorithm does not absolve clinicians from meaningful indication, outcome assessment, and knowledge of clinical exceptions. The clinicians also have to be aware of the follow-up examinations depending on the MELD score and in compliance with the Eurotransplant regulations. [6] For various diseases the lab MELD score does not reflect the patient’s urgency for liver transplant sufficiently. In these cases, the patient is eligible for standard exception (SE) and the match MELD score will be used. The criteria for SE are disease and country specific. The clinical physicians have to be aware of all the applicable regulations. [6] Basis of the match MELD score is the clinical evaluation and diagnostics of the clinical physicians. The match MELD score is not embraced in our system and is subject to patient´s specific discussion in the interdisciplinary liver transplant conference. … Importantly, not knowing relevant patient`s characteristics, the unawareness of the regulations as well as inadvertent mistakes during order creation could lead to an incorrect stated status.”**

**However, we do not agree, that a laboratory specific journal may be a better choice. The project is interdisciplinary and received a great attention starting from the hospital board to Transplant Medicine and Hepatology to Laboratory Medicine.**

Reviewer #2: The significance of this study, and what exactly was accomplished, needs to be more strongly justified. The introduction should provide more clarification as to what kinds of laboratory errors this diagnostic review algorithm is supposed to address.

**Many thanks to your comment on this important aspect. Our system is supposed to identify preanalytical, as well as analytical errors of the MELD diagnostic. ”** **To specify the addressed point we added a more detailed statement on page 7 lines 113ff. “The MELD parameters had to be ordered within the same laboratory order and needed to be send in at the same time. During the process of medical validation, the MELD score and its components were specifically screened for preanalytical errors, such as the plausibility of the dialysis status and the anticoagulation status, confusion of patients, prolonged preanalytical phase and other typical preanalytical errors.**

**After the process of validation, the MELD parameters, as well as the MELD score and the dialysis status were reported in a specific document for further transfer of the data to Eurotransplant. Eurotransplant is responsible for the allocation of donor organs.“ Additionally table 2 provides an impression of typical preanalytical errors that occurred within the MELD diagnostic. However, we decided to include these points within the methods section as it is a major part of the verification algorithm and to avoid unnecessary duplication within the paper.
“Policy violations as occurred in German liver transplant centers in 2012 could have been revealed with very high probability using our specific verification algorithm for MELD diagnostics”** **(page 17 lines 323ff)
Regarding the analytical errors, we described the indeterminable INR within patients with end-stage Liver Disease and discussed the influence of different measuring methods for the parameters on the MELD score. In our opinion, there is still a lack of standardization of the MELD diagnostic to be addressed by the transplant organization in our case Eurotransplant.**

Additionally, while a recent laboratory validation controversy in Germany is mentioned, further support for the need for a validation algorithm would strengthen the study.

**Thank you for this excellent suggestion. We added a paragraph addressing this topic on page 5 lines 76ff. “All German liver transplant centers were reviewed by an external group of specialists from the German Medical Association. Four of the 46 centers failed with serious policy violations, for example dialysis were stated but not performed and blood samples were manipulated. These violations had the consequence that for some patients on the waiting list the priority for liver transplantation was falsely increased.** **To improve the validity of the MELD diagnostics and to prevent irregularities, we implemented a specific quality-ensured lab-MELD diagnostic in 2012 (Fig 1).”**

The manuscript as a whole also would benefit to referring to the "laboratory diagnostic" as a verification algorithm, or some similar terminology, because as it is written, it initially appeared that the study was on a new form of laboratory test that somehow calculates MELD directly from a sample.

**Thank you. We changed the terminology at the suitable sections within the whole paper. We hope the change of our terminology helps to increase the comprehensibility of our paper. We now refer to our system as “verification algorithm for MELD diagnostic”.**

A more granular and clearly labeled diagram of the verification algorithm should be included. It is hard to tell what types of findings would trigger review by the laboratory medicine group in this "diagnostic", and therefore these findings are, as yet, not applicable to other centers. As this is an implementation/quality improvement type project, a clear algorithm would be helpful, or at least examples: for example, where you state that "conspicuous constellations" in Figure 1 trigger a review, suggest some of these conspicuous constellations.

**Thank you. We agree that concretization of conspicuous constellations are helpful to reproduce our system in other transplantation centers. However, we think additional information about those conditions within the figure would reduce the figure’s clarity. We decided to concretize this aspect in the subtext of figure 1. “Conspicuous or implausible constellations may be: deviation in comparison to previous analysis, remarkably changes in creatine or bilirubin concentration, INR values, preanalytical errors as hemolysis or extended time spans between different materials of the same order or between test ordering and arrival at the laboratory. Implausible constellations are checked** **by personnel specifical trained in laboratory medicine in the context of other liver synthesis parameters such as fibrinogen, albumin or the choline esterase rate.”**

Page 11, line 105: "blood takings" should be "blood draws" or "blood samples needed to be procured". Line 111 on the same page should have "the" before "ethics committee". Note that in line 163, you state there were cases in which further blood draws were performed if the original sample was inadequate; please change line 105's statement to reflect that this study did not require further blood draws beyond those needed for adequate MELD determination.

**Thank you for these corrections. We changed the points in the manuscript at the corresponding place.**

6. PLOS authors have the option to publish the peer review history of their article ([what does this mean?](https://journals.plos.org/plosone/s/editorial-and-peer-review-process#loc-peer-review-history)). If published, this will include your full peer review and any attached files.


If you choose “no”, your identity will remain anonymous but your review may still be made public.


Do you want your identity to be public for this peer review? For information about this choice, including consent withdrawal, please see our [Privacy Policy](https://www.plos.org/privacy-policy).

Reviewer #1: No

Reviewer #2: No
