# Supplementary material for: Experiences from six years of quality assured Model of End Stage Liver Disease (MELD) diagnostics
Source: PLoS One. 2021 Aug 26;16(8):e0254219. doi: 10.1371/journal.pone.0254219 (PMC8389365; doi:10.1371/journal.pone.0254219)
Supplement: Attachment — Submitted filename: 210601_PLOSONE_rebuttal_letter.docx [file pone.0254219.s003.docx]

Reviewer's Responses to Questions

**Comments to the Author**
1. If the authors have adequately addressed your comments raised in a previous round of review and you feel that this manuscript is now acceptable for publication, you may indicate that here to bypass the “Comments to the Author” section, enter your conflict of interest statement in the “Confidential to Editor” section, and submit your "Accept" recommendation.

Reviewer #2: (No Response)

Reviewer #3: All comments have been addressed

2. Is the manuscript technically sound, and do the data support the conclusions?

The manuscript must describe a technically sound piece of scientific research with data that supports the conclusions. Experiments must have been conducted rigorously, with appropriate controls, replication, and sample sizes. The conclusions must be drawn appropriately based on the data presented.

Reviewer #2: Yes

Reviewer #3: Yes

3. Has the statistical analysis been performed appropriately and rigorously?

Reviewer #2: Yes

Reviewer #3: Yes

4. Have the authors made all data underlying the findings in their manuscript fully available?

The [PLOS Data policy](http://www.plosone.org/static/policies.action#sharing) requires authors to make all data underlying the findings described in their manuscript fully available without restriction, with rare exception (please refer to the Data Availability Statement in the manuscript PDF file). The data should be provided as part of the manuscript or its supporting information, or deposited to a public repository. For example, in addition to summary statistics, the data points behind means, medians and variance measures should be available. If there are restrictions on publicly sharing data—e.g. participant privacy or use of data from a third party—those must be specified.

Reviewer #2: Yes

Reviewer #3: Yes

5. Is the manuscript presented in an intelligible fashion and written in standard English?

PLOS ONE does not copyedit accepted manuscripts, so the language in submitted articles must be clear, correct, and unambiguous. Any typographical or grammatical errors should be corrected at revision, so please note any specific errors here.

Reviewer #2: Yes

Reviewer #3: Yes

6. Review Comments to the Author

Please use the space provided to explain your answers to the questions above. You may also include additional comments for the author, including concerns about dual publication, research ethics, or publication ethics. (Please upload your review as an attachment if it exceeds 20,000 characters)

Reviewer #2: Line 22: add “the” before “laboratory MELD diagnostic” or make diagnostics plural, for appropriate grammar
Lines 78-79: correct conventions such as follows: “policy violations; for example, dialysis was stated but not performed, or blood samples were manipulated.”
Lines 79-81: correct conventions such as follows: “These violations had the consequence that for some patients on the waiting list, priority for liver transplantation was falsely increased.”
Lines 90 to 96: Change “constellations may be” to “may include”; change “remarkably” to a more concrete term such as “improbable” or “extreme.”

**Thank you for these corrections. We changed the points in the manuscript at the corresponding place.**

Also, what does “materials of the same order” mean? Do you mean “components”?

**Thank you. For the correct calculation of the MELD score a citrate blood tube and a serum tube is needed. Both materials must be sent in at the same time within the same laboratory order. We changed the terminology to “components”, and we hope it will be more understandable.**

Change “specifical” to “specifically”. Also, use semi-colons instead of commas to separate list items when list items contain a list – i.e., “deviation in comparison to previous analysis; improbable changes in creatine, bilirubin concentration, or INR values; and preanalytical errors such as hemolysis, or extended time spans between different materials of the same order or between test ordering and arrival at the laboratory.”
Lines 116-117: “confusion of patients” should be restated. I believe you are referring to misidentification of patients – rather than confusion as a mental status.
Lines 123-124: Restate “A notification during the order creation informed ensured the use for this indication.” It appears informed is an extra word here.

**Thank you for these corrections. We changed the points in the manuscript at the corresponding place.**
Table 2: Does “missing material” refer to a missing blood draw, an incorrect tube used, or something else?

**Thank you. For the correct calculation of the MELD score a citrate blood tube and a serum tube is needed. As both materials must be sent in at the same time within the same laboratory order, just one of the materials was sent in, resulting in “missing material”. We corrected it within the table for improved clarity.**

Again, as above, use “misidentification” rather than “confusion” to refer to incorrect patient ID
Lines 258-259: Add “the” before “basis”
Line 260: replace the word “embraced” with something more clinical such as “incorporated”; add “a” or “the” before “patient’s specific”
Line 265: add “the” before “relevant patient’s”

**Thank you for these corrections. We changed the points in the manuscript at the corresponding place.**

Reviewer #3: This is a revised version of a paper that I did not originally review. The information provided is of interest. The authors have addressed all previous comments in a satisfactory way.

**Thank you for your review.**

7. PLOS authors have the option to publish the peer review history of their article ([what does this mean?](https://journals.plos.org/plosone/s/editorial-and-peer-review-process#loc-peer-review-history)). If published, this will include your full peer review and any attached files.


If you choose “no”, your identity will remain anonymous but your review may still be made public.


**Do you want your identity to be public for this peer review?** For information about this choice, including consent withdrawal, please see our [Privacy Policy](https://www.plos.org/privacy-policy).

Reviewer #2: No

Reviewer #3: No
